# A Novel Injectable Calcium Phosphate Cement-Bioactive Glass Composite for Bone Regeneration

**DOI:** 10.1371/journal.pone.0062570

**Published:** 2013-04-25

**Authors:** Long Yu, Yang Li, Kang Zhao, Yufei Tang, Zhe Cheng, Jun Chen, Yuan Zang, Jianwei Wu, Liang Kong, Shuai Liu, Wei Lei, Zixiang Wu

**Affiliations:** 1 Institute of Orthopedics, Xijing Hospital, The Fourth Military Medical University, Xi’an, Shaanxi Province, People’s Republic of China; 2 School of Materials and Engineering, Xi’an University of Technology, Xi’an, Shaanxi Province, People’s Republic of China; 3 Department of Oral and Maxillofacial Surgery, School of Stomatology, The Fourth Military Medical University, Xi’an, Shaanxi Province, People’s Republic of China; University of California, San Diego, United States of America

## Abstract

**Background:**

Calcium phosphate cement (CPC) can be molded or injected to form a scaffold in situ, which intimately conforms to complex bone defects. Bioactive glass (BG) is known for its unique ability to bond to living bone and promote bone growth. However, it was not until recently that literature was available regarding CPC-BG applied as an injectable graft. In this paper, we reported a novel injectable CPC-BG composite with improved properties caused by the incorporation of BG into CPC.

**Materials and Methods:**

The novel injectable bioactive cement was evaluated to determine its composition, microstructure, setting time, injectability, compressive strength and behavior in a simulated body fluid (SBF). The in vitro cellular responses of osteoblasts and in vivo tissue responses after the implantation of CPC-BG in femoral condyle defects of rabbits were also investigated.

**Results:**

CPC-BG possessed a retarded setting time and markedly better injectability and mechanical properties than CPC. Moreover, a new Ca-deficient apatite layer was deposited on the composite surface after immersing immersion in SBF for 7 days. CPC-BG samples showed significantly improved degradability and bioactivity compared to CPC in simulated body fluid (SBF). In addition, the degrees of cell attachment, proliferation and differentiation on CPC-BG were higher than those on CPC. Macroscopic evaluation, histological evaluation, and micro-computed tomography (micro-CT) analysis showed that CPC-BG enhanced the efficiency of new bone formation in comparison with CPC.

**Conclusions:**

A novel CPC-BG composite has been synthesized with improved properties exhibiting promising prospects for bone regeneration.

## Introduction

Calcium phosphate biomaterials, such as hydroxyapatite (HA) ceramic, calcium phosphate ceramics and calcium phosphate cements (CPC), have been widely used as bone substitute materials in clinical applications due to their good biocompatibility and osteoconduction [1]. However, it is difficult to fill irregularly shaped bone defects with sintered bioactive ceramics and these materials have obvious limitations for use in minimally invasive surgery [2]. CPC can be molded or injected to form a scaffold in situ that intimately conforms to the shape of complex bone defects [3]. In 1986, a typical CPC composed of a powdered mixture of tetracalcium phosphate (TECP) [Ca_4_ (PO_4_)_2_O] and dicalcium phosphate anhydrous (DCPA) (CaHPO_4_) was first reported by Brown and Chow [4]. This CPC powder could be mixed with aqueous liquid to form a paste that would set in situ and form HA (Ca_10_ (PO_4_)_6_ (OH)_2_) as a final product, as the main constituent part of the mineral phase of bone [5]. Due to its high biocompatibility, osteoconduction and bone replacement capability, CPC was approved in 1996 by the Food and Drug Administration (FDA) to repair craniofacial defects [6]. Since then, several other calcium phosphate cements and injectable cements have been developed [7], [8]. However, under adverse critical conditions, such as poorly vascularized sites and elderly patients with metabolic disorders, the osteoconductive and degradation properties of CPC are not sufficient to achieve complete bone regeneration. Hence, it is necessary to enrich CPC with osteopromotive or osteoinductive factors to improve its biological performance [9].

As a surface-active bone substitute, bioactive glass (BG) has recently attracted attention due to its good biocompatibility both in bone and in soft tissues [10]. Bioactive glasses e.g. 45S5 bioactive glass bond strongly to bone and promote bone growth [11] by forming a hydroxycarbonate apatite (HCA) layer and releasing Ca, P and Si ions [10]. These ions are supposed to stimulate osteogenesis [12]. 45S5 bioactive glass promoted the attachment, proliferation, differentiation and mineralization of osteoblast-like cells [13]; up-regulated seven families of osteoblast genes in osteoprogenitor cells; increased osteoblast phenotype expression of osteoprogenitor cells; [14] and induced differentiation of bone marrow stromal cells into mature osteoblasts [15], suggesting that this bioactive glass creates both solution-mediated and surface-mediated effects on bone cell activity. However, previous studies have mainly focused on the preparation of BG in the form of a scaffold. Relevant literature only recently became available regarding CPC-BG as applied in minimally invasive injectable grafts [16].

The purpose of the present study was to synthesize a type of self-setting bioactive cement by the incorporation of BG into CPC. The composition, morphology/microstructure, setting time, injectability, compressive strength, surface reaction layer formation and degradation of CPC-BG were investigated, and the cell and tissue responses to CPC-BG were also investigated both in vitro and in vivo.

## Materials and Methods

### Preparation and Characterization of the CPC-BG

CPC consisted of a powder and a liquid phase. The CPC powder was composed of tetracalcium phosphate (TECP) and dicalcium phosphate anhydrous (DCPA) in an equal molar ratio, and the preparation method was as previously described [17]. Briefly, TECP was synthesized by a solid-to-solid reaction between calcium phosphate and calcium carbonate at a temperature of 1500°C for 8 h. Dicalcium phosphate dehydrate (DCPD, CaHPO_4_·2H_2_O) was prepared from ammonium hydrogen phosphate ((NH_4_)_2_HPO_4_) and calcium nitrate (Ca(NO_3_)_2_) in an acidic environment. DCPA was obtained by removing the crystallization water in DCPD at 120°C. The TECP and DCPA powders were then mixed in a micromill to form the CPC powder. All the chemicals used were purchased from Sinopham Chemical Reagent Co. Ltd.

Bioglass 45S5 (Wt %: 45% SiO_2_, 24.5% Na_2_O, 24.5% CaO and 6% P_2_O_5_) were provided by NovaBone® (LLC, Alachua, USA). The NovaBone® product were ground in a ball mill and sieved to obtain 45S5 particles with sizes ranging from approximately 5–10 µm, with a median of 6 µm.

The CPC-BG powder was prepared by adding BG 45S5 powder (10 and 20 wt%) into the CPC powder. The CPC-BG composite powders were mixed with potassium phosphate buffers (pH 7.0) for 1 min at the given P/L ratio (2.0 g/mL) with a spatula to form homogeneous paste. The paste was then placed into a plastic cylindrical mold with a diameter of 5 mm and a height of 10 mm for mechanical testing, a circular mold with a diameter of 10 mm and a thickness of 3 mm for measurement of in vitro bioactivity, degradability and cell studies. Each specimen was set in a 100% relative humidity box at 37°C for 24 h, at which time the hardened CPC-BG composite was obtained. X-ray diffraction (XRD, Dandong fangyuan Co., China) with CuKα radiation in a continuous scan mode was adopted to characterize the phase composition of the phase composition of the specimens. The 2θ range was from 10° to 90° at a scanning speed of 2.4°/min. The cross-section of the specimens was examined with a scanning electron microscopy (SEM, Hitachi S-4800, Japan) equipped with an energy dispersive spectrometer (EDS, Falcon, USA).

### Setting time, Injectability and Compressive Strength of CPC-BG

CPC-BG paste was placed into a plastic cylindrical mold with a diameter of 5 mm and a height of 10 mm, and it was then allowed to set in a 100% relative humidity box at 37°C.

The setting time was taken as the time at which the paste hardened to such an extent that a needle (300 g, Φ = 1 mm) would not penetrate deeper than 1 mm into the sample. This criterion to determine the setting time is based on the American Society for Testing and Materials C 187-98 standard test method for normal consistency of hydraulic cement, which is also called the Vicat method, to determine the setting time. Each specimen was performed in triplicate and the average value was calculated.

The injectability of CPC-BG composite paste was evaluated by extruding 2.0 g of as-prepared paste through a 2.5 mL disposable syringe with an opening nozzle with the diameter of 2.0 mm by hand, according to a modified method described previously [18], suggesting that injection by hand possessed even slightly lower standard deviations than injection by machine with preset load. After setting at 37°C in a 100% relative humidity box for pre-selected time, the paste was extruded from the syringe until it was unable to be injected. The weight of the paste injected through the syringe was measured. The injectability was calculated as: I = m_ injected_/m_ initial_×100%, where I is the injectability, m_ injected_ and m_ initial_ are the weight of the paste injected through the syringe and the paste initially contained in the syringe. All values were the average of three tests performed for each group and presented as mean ± standard deviation (mean ± SD).

After hardened at 37°C in a 100% relative humidity box for 1 and 7 days, the compressive strength of CPC-BG composite specimens was measured at a loading rate of 1 mm/min with a universal testing machine (MTS-858, MTS System Inc, USA). The compressive strength was calculated as following: S = Fmax/A, where Fmax is the maximum load on the load-deformation curve and A is the cross-sectional area of each specimen. The measurement was performed three times and the results were expressed as mean ± SD.

### Bioactivity and Degradation in Simulated Body Fluid (SBF)

Simulated body fluid (SBF), which has ion concentrations and a pH value similar to those of human blood plasma, was prepared in accordance with the procedure described [19]. After setting for 24 h, the paste specimens (10 mm diameter and 3 mm thickness) were soaked in an SBF solution at 37°C for 7 and 14 days with a weight-to-volume ratio of 0.2 g/ml and the solution was refreshed every day. For the evaluation of in vitro bioactivity, the samples were removed after incubation for specified time periods, rinsed in deionized water and dried at room temperature until a constant weight was attained. The specimens were characterized with EDS, and the surface morphologies of the specimens were observed with SEM.

For the measurement of in vitro degradation, the 7-day-set paste specimens were immersed into SBF solution at 37°C for 28 days with a weight-to-volume ratio of 0.2 g/ml and the solution was refreshed every day. For this evaluation, the samples were removed after incubation for specified time periods, rinsed in deionized water and dried at 60°C for 24 h and weighed. In vitro degradation was measured as D = [(W_0_−Wt)/W_0_]×100%, where D is the degradation rate and W_0_ and Wt are the dry weight of the initial specimen and the degraded specimen, respectively. All values presented are the average of five tests performed for each sample.

### Cell Attachment, Proliferation, Morphology and Differentiation

With protocols approved by the Institutional Animal Care Committee of Xijing Hospital (Permit Number: 08–269), osteoblasts were obtained from calvariae of 1-to 2-day-old Sprague-Dawley rats with an enzyme-digestion technique as previously described [20]. Briefly, under general anesthesia, calvariae were dissected aseptically, rinsed with PBS several times, stripped of the periosteum and adherent tissue and then minced. The minced fragments were incubated with 4 ml of 0.25% trypsin (Sigma, USA) for 20 min with gentle shaking at room temperature. Then, the trypsin was removed and fetal bovine serum (FBS, HyClone, USA) was added to terminate digestion. After digestion, the fragments were uniformly attached to each culture flask bottom and were cultured in a humidified atmosphere of 5% CO_2_ at 37°C. After 24 h, Dulbecco’s modified Eagle’s medium (DMEM, Sigma, USA) supplemented with 1% penicillin/streptomycin antibiotics (Sigma, USA) and 10% FBS was added to the flasks. The medium was changed every 2 days. Cell subcultures at passage 2 were used in the following studies.

To investigate cell attachment and proliferation, the CPC and CPC-BG composite specimens (10 mm diameter and 3 mm thickness) were sterilized by ^60^Co irradiation for 12 h. Prior to cell seeding, CPC and CPC-BG composite specimens were pre-wetted with basal tissue culture medium (DMEM supplemented with 1% penicillin/streptomycin antibiotics and 10% FBS) after setting for 24 h. Osteoblasts with a density of 5×10^4^ cells/well were seeded onto the specimens. Cell attachment study was determined with a 3-(4, 5-dimethylthiazol-2-yl)-2, 5-diphenyl tetrazolium bromide (MTT) (Sigma, USA) assay. The specimen-cell constructs were incubated in a humidified atmosphere of 5% CO_2_ at 37°C for 4, 8 h, rinsed in 0.15 M PBS, immersed in a mixture of serum-free cell culture medium and MTT reagent (5∶1), followed and incubated in a humidified atmosphere of 5% CO_2_ at 37°C for 4 h. The supernatant from each well was then carefully removed and dimethyl sulfoxide was added to ensure the solubilization of crystals. Next, 150 µl of the reaction solution with the cells was carefully transferred to 96-well plates, and the optical density (OD) values at 492 nm were measured. Six specimens in each group were tested for each incubation period; each test was carried out in triplicate per specimen. The results were presented as means ± SD.

Cell proliferation was evaluated by seeding cells at a density of 5×10^4^ cells per sample followed by incubation for 1, 4 and 7 days; the medium was replaced every second day. Adhesion and cell viability on the substrates were assessed quantitatively using the MTT assay. Six specimens in each group were tested for each incubation period; each test was carried out in triplicate per specimen. The results were presented as means ± SD.

Cell attachment and morphology were confirmed by direct visualization of specimen-cell constructs under SEM. The cells were attached to the specimens for 1 and 4 days in a humidified atmosphere of 5% CO_2_ at 37°C. At a pre-selected time point, the specimen-cell constructs were removed, rinsed with PBS twice and fixed with 2.5% glutaraldehyde in 0.1 M PBS for 30 min. The fixed constructs were washed with PBS three times, dehydrated in graded ethanol, vacuum-dried at 37°C overnight and sputter-coated with gold-palladium prior to SEM observation.

To evaluate ALP activity, the medium was aspirated and specimens were moved to new 24-well plates after 4 and 7 days of incubation. Approximately 500 µl of cell lysis buffer containing Triton X-100 was added to each well at room temperature to lyse the cells. The cell lysate was placed in a 1.5 mL centrifuge tube, centrifuged and then frozen to −20°C. At 4 and 7 days, the frozen samples were thawed at room temperature for 5 min to measure ALP activity following the manufacturer’s instructions (Sigma, USA). The absorbance of ALP was quantified with a plate reader at 405 nm. The total protein content was determined by the bicinchoninic acid method using a Pierce protein assay kit (Pierce Biotechnology Inc., USA) according to the manufacturer’s instructions. The ALP activities were normalized to the total protein content. Each experiment was carried out in triplicate, and the results were presented as means ±SD.

### Implantation in vivo

Eighteen healthy female New Zealand white rabbits aged 4–5 months and weighing 2.5–3 kg were randomly divided into three groups of 6 animals for each type of implant. The experiment was carried out in strict accordance with the recommendations in the Guide for the Care and Use of Laboratory Animals for the National Institutes of Health. The animal protocol was approved by the Institutional Animal Care Committee of Xijing Hospital (Permit Number: 08–269). Under general anesthesia and sterile conditions, cylindrical specimens (6 mm diameter and 10 mm height) were implanted into each femoral condyle. Then, the wounds were sutured and penicillin (240, 000 UI) was injected into the rabbits for 3 days. Three animals of each group were sacrificed with an overdose abdominal injection of pentobarbital sodium at 4 and 12 weeks after implantation. The bone specimens were harvested immediately after sacrifice and fixed in 10% neutral buffered formalin. The macroscopic appearance of the defects was evaluated to assess the degree of specimen incorporation and tissue reactions adjacent to the specimens. For the micro-computed tomography (micro-CT) analysis, the bone specimens were imaged with three-dimensional microfocus computed tomography (micro-CT, eXplore Locus SP, GE, USA) at a voltage of 80 kVp and an electric current of 80 mA. To evaluate the in vivo resorption of the implanted materials, the residual material volume fraction (RMVF) was calculated as RMVF = VR/VT where VR is the volume of residual material and VT is the total volume of material. The new bone volume was quantified as the bone volume fraction (BVF) with the formula BVF = VB/VT where VB is the newly formed bone volume and VT is the total material volume. For histological evaluation, the bone specimens were embedded in methacrylate resin after micro-CT scanning. Tissue blocks were sectioned to 5 µm thickness, and at least three slices of histology sections were randomly obtained. The sections were then stained with Van Gieson’s Stain and observed with a light microscope (Nikon Microphot FXA).

### Statistical Analysis

Experimental data were expressed as means ± SD and the Student’s *t*-test or one-way analysis of variance (ANOVA) with post hoc tests was applied to comparison. Differences were considered statistically significant at *p*<0.05.

## Results

### Characterization of CPC-BG

**Figure 1:**
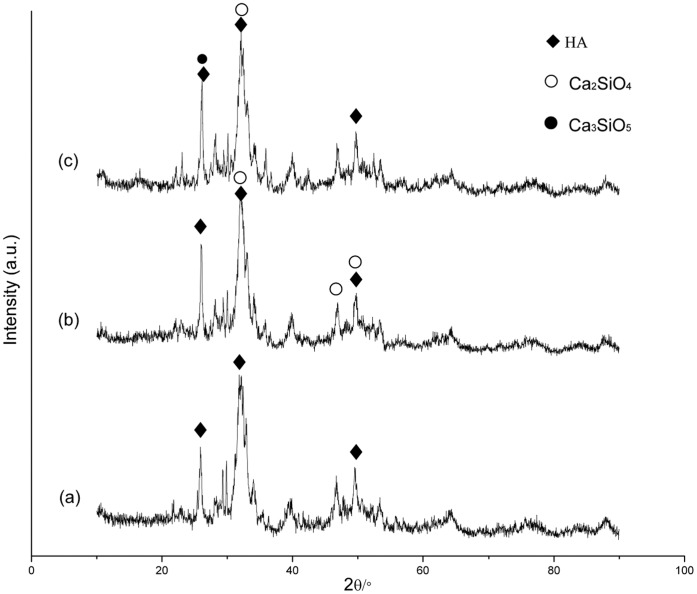
X-ray diffraction patterns of the CPC and CPC-BG composite specimens after setting for 24 h. (a) CPC. (b) CPC+10% BG. (c) CPC+20% BG.

After setting for 24 h in a 100% relative humidity box at 37°C, XRD showed that the hardened CPC contained diffraction peaks of HA. The main peaks for the HA of a hardened CPC-BG composite, were not obviously altered and peaks for Ca_2_SiO_4_ could be seen in the XRD patterns of the CPC-BG composite with 10% BG (Figure 1). Moreover, the presence of Ca_2_SiO_4_ and Ca_3_SiO_5_ within the CPC-BG composite containing 20% BG was also confirmed by XRD patterns.

**Figure 2:**
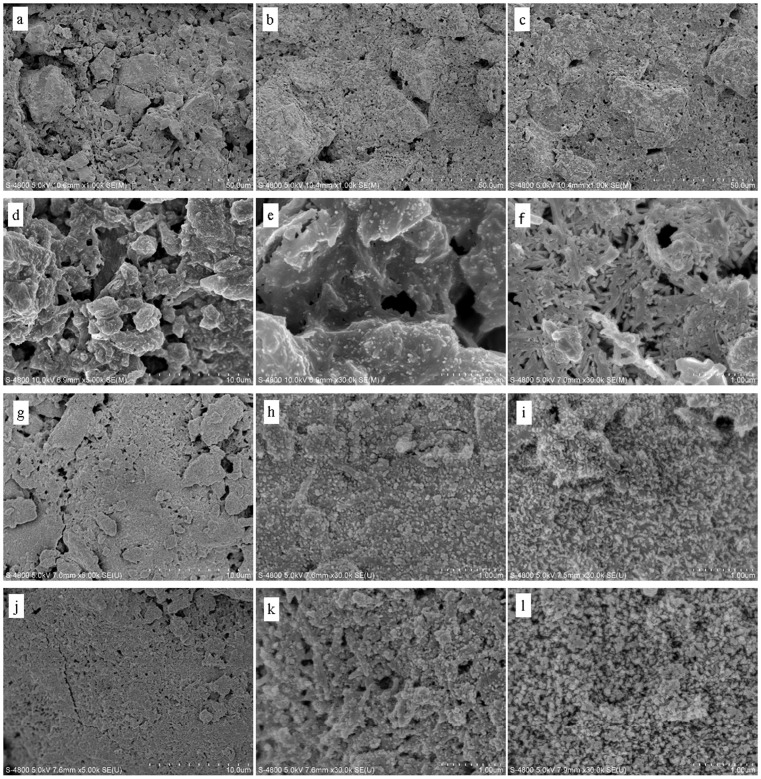
SEM micrographs of cross-sections of CPC and CPC-BG composite specimens that were hardened for 24 h (a–c) and specimens immersed in SBF for different times (d–l). (a) CPC. (b) CPC+10% BG. (c) CPC+20% BG. (d, e) CPC after 7 days. (f) CPC after 14 days. (g, h) CPC+10% BG after 7 days. (i) CPC+10% BG after 14 days. (j, k) CPC+20% BG after 7 days. (l) CPC+20% BG after 14 days.

The SEM micrographs for the cross section of the CPC and CPC-BG composite specimens (10% and 20% BG) showed that the CPC-BG composite specimens closely combined with each other and showed more compact microstructure than CPC alone, while the CPC formed a clay-like structure with many micropores, after setting for 24 h in a 100% relative humidity box at 37°C (Figure 2a–2c). Moreover, the microstructure of the hardened CPC-BG composite specimens became more compact with the increased BG content.

### Setting Time, Injectability and Compressive Strength

For all CPC-BG composite pastes, the setting times were prolonged as the content of BG increased; the time increased from 21 min to 25 min when the weight ratio of BG varied from 10% to 20% at a P/L ratio of 2.0 g/ml (Table 1).

**Table 1:** The setting time of the cement pastes (P/L = 2.0 g/ml).

	CPC	CPC+10% BG	CPC+20% BG
Setting time (min)	15±0.8	21±1.4	25±0.9

**Figure 3:**
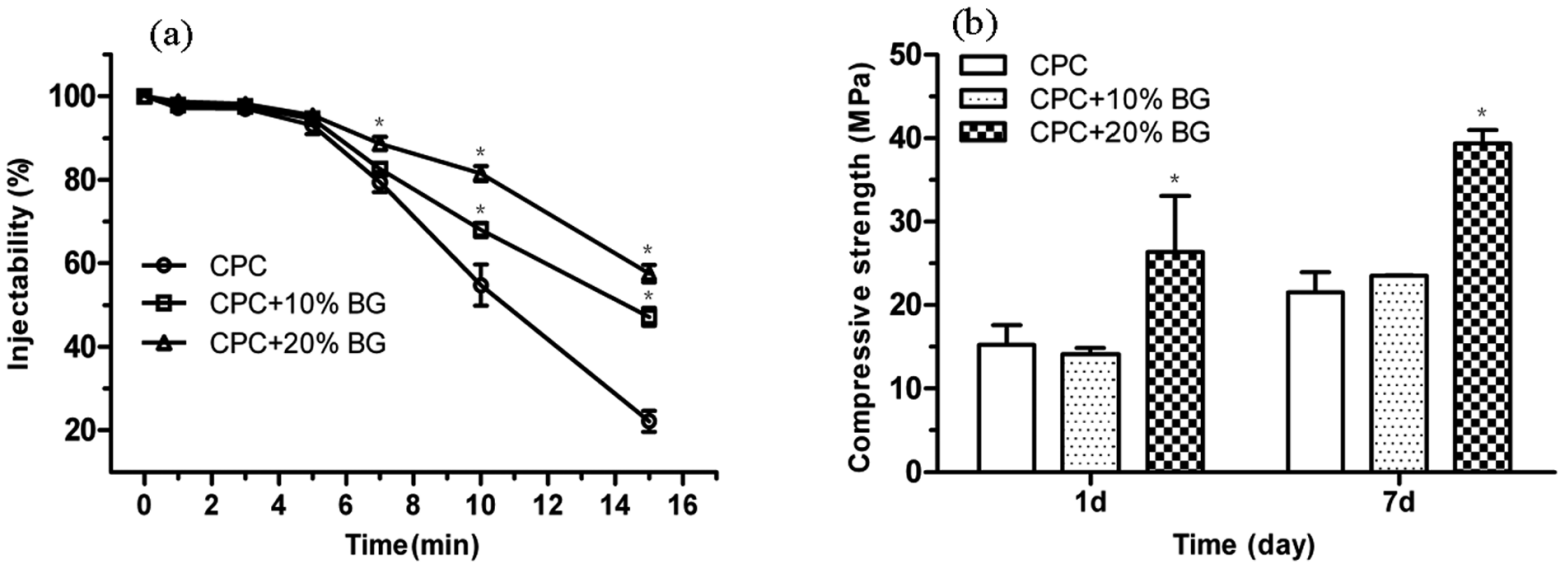
Injectability of the CPC and CPC-BG composite pastes versus setting time (P/L = 2.0 g/ml) (a) and compressive strength of the CPC and CPC-BG composite specimens after setting for 1 and 7 days (P/L = 2.0 g/ml) (b). An asterisk (*) indicates that the injectability and compressive strength of the CPC-BG composite specimens were significantly different from those of CPC (*p*<0.05).

The injectability of the CPC-BG composite paste was significantly improved compared with the injectability of the CPC paste (Figure 3a). Moreover, the CPC-BG composite paste did not give any demixing when the weight ratio of BG increased from 10% to 20% due to the filter-pressing effect during extrusion through the syringe. Furthermore, the injectability of the CPC-BG composite paste rose with an increase in BG content.

After setting in a 100% relative humidity box at 37°C for 1 and 7 days, the compressive strength of CPC and CPC-BG composite specimens increased with prolonged time during setting (Figure 3b). Furthermore, the compressive strength of the CPC-BG composites rose with increasing BG weight ratio. The composite with 20% BG exhibited the highest compressive strength after setting for 7 days. The compressive strength of CPC-BG composites (20%) reached 26 MPa at 1 day and 40 MPa at 7 days compared with only 15 MPa and 22 MPa of CPC at the same time points, respectively. There were significant differences between CPC and CPC-BG composite (20%) specimens at day 1 and day 7 (*p*<0.05).

### Bioactivity and Degradation in SBF

After soaking for 7 days, the nano-sized aggregates of the bone-like apatite appeared on both surfaces of both CPC and CPC-BG composite specimens (Figure 2d–2l). With longer immersion time, the amount and grain size of apatite particles on the CPC and CPC-BG composite surfaces increased; thus, the apatite layers increased in density. However, the apatite aggregates on the CPC-BG composite surface were larger in number and denser than those on CPC surface after immersion for 7 and 14 days. Moreover, many crystals formed agglomerates and further congregated to form a layer on the surface of the CPC-BG composite and there was a noticeable increase in the density of the apatite structures with the increase of BG content.

**Figure 4:**
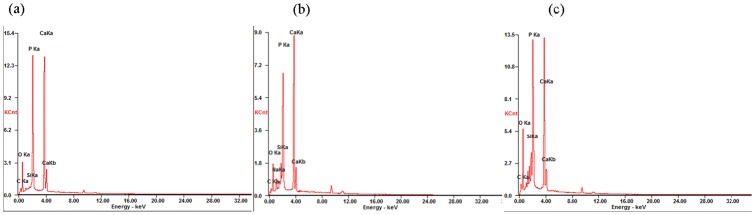
EDS analysis of the CPC and CPC-BG composite specimens immersed in SBF for 7 days. (a) CPC. (b) CPC+10% BG. (c) CPC+20% BG.

EDS indicated that the surfaces of the CPC-BG composites (10% and 20%) consisted mainly of a calcium phosphate with Ca/P ratios of approximately 1.56 and 1.53, respectively with Si and some Na and Mg ions from soaking in the SBF solution for 7 days. Conversely, the surface of CPC consisted of a calcium phosphate with a Ca/P ratio of approximately 1.67, and it contained no Si but did have some Na and Mg ions from the SBF solution (Figure 4).

**Figure 5:**
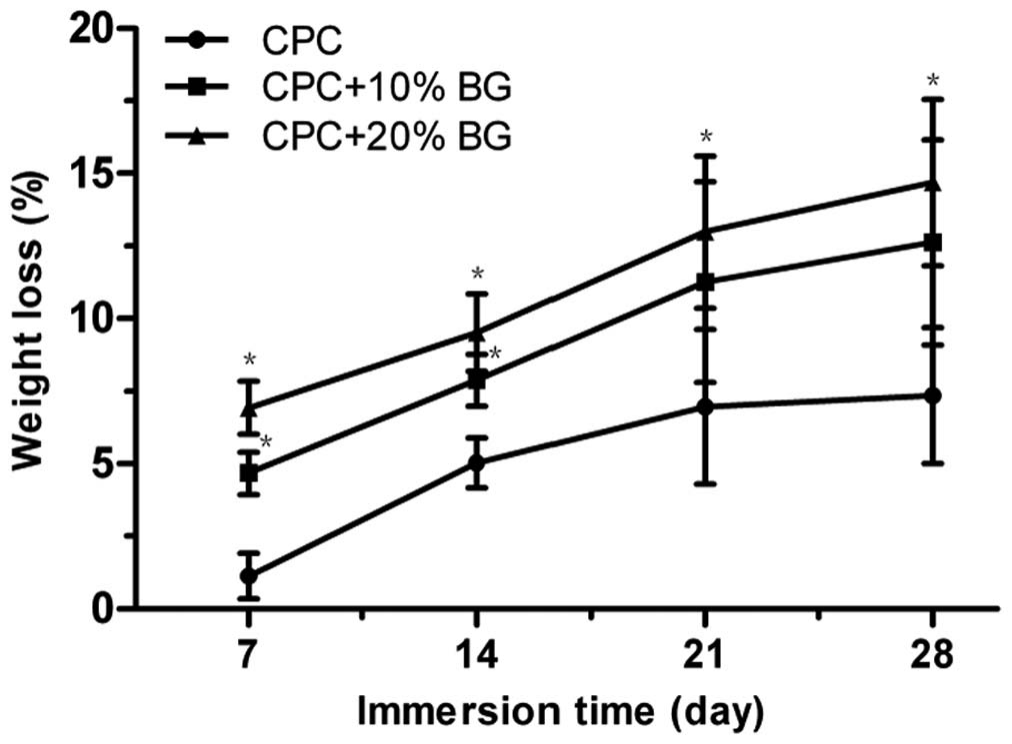
Weight-loss ratios of the CPC and CPC-BG composite specimens immersed in SBF for various periods. An asterisk (*) indicates that the weight-loss ratio of the CPC-BG composite specimens was significantly different than that of CPC (*p*<0.05).

The degradation rates of the specimens were characterized by their weight loss ratios in SBF solution. After soaking in SBF solution at 37°C for 28 days, the degradation rates of all CPC-BG composite specimens were significantly higher than the degradation rates of CPC specimens from 7 to 14 days (*p*<0.05).The degradation rates of CPC-BG composite specimens (20%) were significantly higher from 21 to 28 days compared with the degradation rates of CPC specimens (*p*<0.05) (Figure 5). Furthermore, the degradation rate rose with an increase in BG content.

### Cell Attachment, Proliferation, Morphology and Differentiation

**Figure 6:**
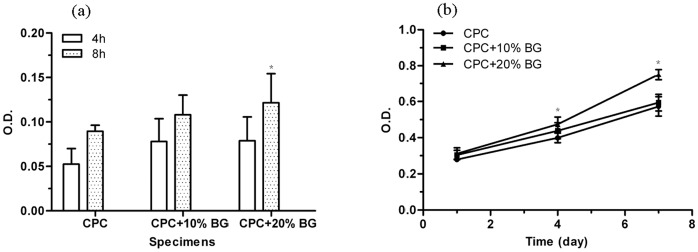
Cell attachment on CPC and CPC-BG composite specimens at 4 h and 8 h (a), Cell viability on CPC and CPC-BG composite specimens after 1, 4 and 7 days (b). An asterisk (*) indicates that the cell attachment and proliferation of CPC-BG composite specimens were significantly different from those of CPC (*p*<0.05).

The MTT assay was adopted to assess the number of cells that adhered to the various biomaterials because OD absorbance values can be used as an indicator of the number of cells. There were no significant differences at 4 h for all the specimens (Figure 6a). However, the OD values of the CPC-BG composite specimens (20%) were significantly higher than the OD values of the CPC specimens (*p*<0.05) after a period of 8 h. There was no significant difference between CPC and CPC-BG composite (10%) although the OD values of CPC-BG composite (10%) were higher than the OD values of cells on CPC. In addition, the OD values of the CPC-BG composite rose with an increase in BG content and the highest OD values were obtained in the composite with 20% BG after 8 h.

The viability of osteoblasts cultured on CPC and CPC-BG composite specimens was assessed with the MTT assay because the OD values can provide an indication of cell growth and proliferation on various biomaterials. The OD values of the CPC-BG composite specimens (20%) were significantly higher than the OD values of the CPC specimens after 4 and 7 days (*p*<0.05), indicating that the CPC-BG composite specimens promoted cell growth and facilitated proliferation with no cytotoxic effect on cells compared with CPC specimens **(** Figure 6b).

**Figure 7:**
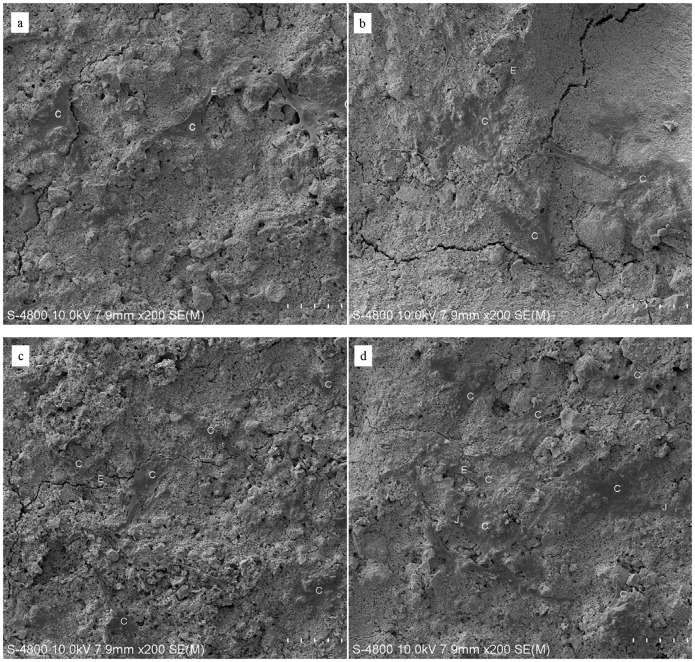
SEM micrographs of the morphological features of cells cultured on the CPC and CPC-BG composite specimens for 1 and 4 days. Images of cells cultured on (a) CPC and (b) CPC-BG composite specimens for 1 day. Images of cells cultured on (c) CPC and (d) CPC-BG composite specimens for 4 days. C: cells. E: Cytoplasmic extensions of the cells. J: cell-cell junctions.

Cells firmly attached and spread well on the surface of CPC and CPC-BG composite specimens with morphologically normal appearance after for 1 day of culture (Figure 7a, b). As number of cells increased, the cells extended, spread well and exhibited intimate attachment to the surfaces of the CPC and CPC-BG composite specimens with cytoplasmic extensions after 4 days (Figure 7c, d). Cell-to-cell junctions appeared in the SEM images. The number of cells on the CPC-BG composite specimens was greater than the amount on the CPC specimens.

**Figure 8:**
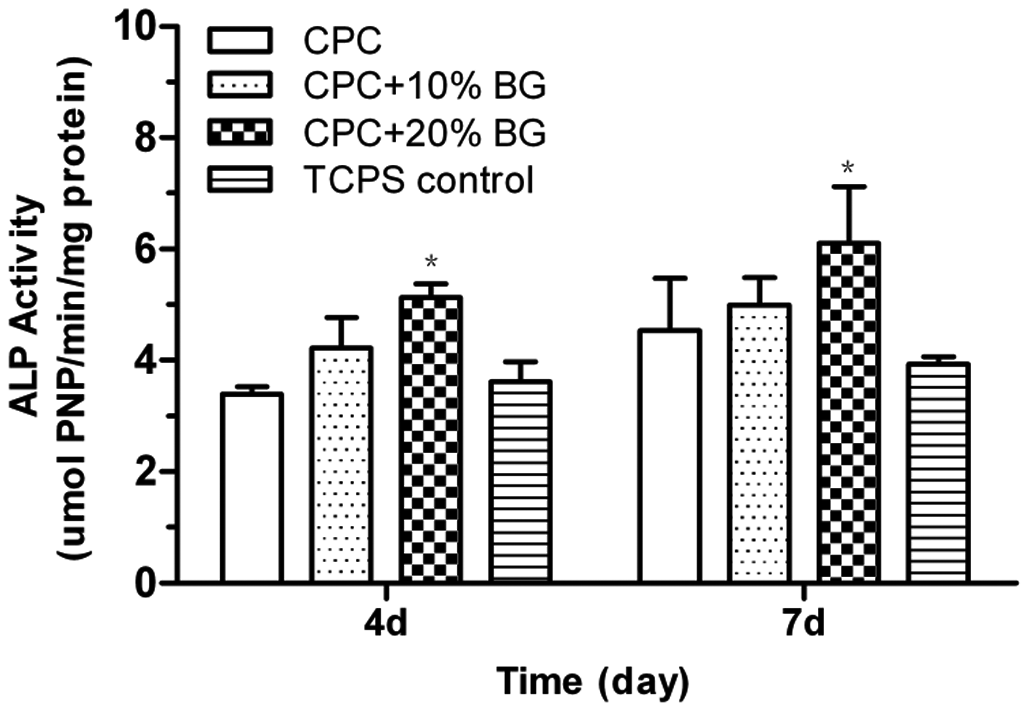
Alkaline phosphatase activity (ALP) of cells cultured on the CPC and CPC-BG composite specimens for 4 and 7 days with tissue-cultured polystyrene (TCPS) as the control. An asterisk (*) indicates that the ALP activities of cells cultured on CPC-BG composite specimens were significantly different from those of CPC and the TCPS control (*p*<0.05).

Cell differentiation was evaluated by testing the ALP activity of rat osteoblasts cultured on CPC and CPC-BG composite specimens after 4 and 7 days. The ALP activity of cells cultured on the CPC-BG composite (20%) was significantly higher than the ALP activity on the CPC and TCPS controls (*p*<0.05) after 4 and 7 days (Figure 8). Moreover, there was no significant difference among CPC, CPC-BG composite (10%), and TCPS control.

### Macroscopic Evaluation

**Figure 9:**
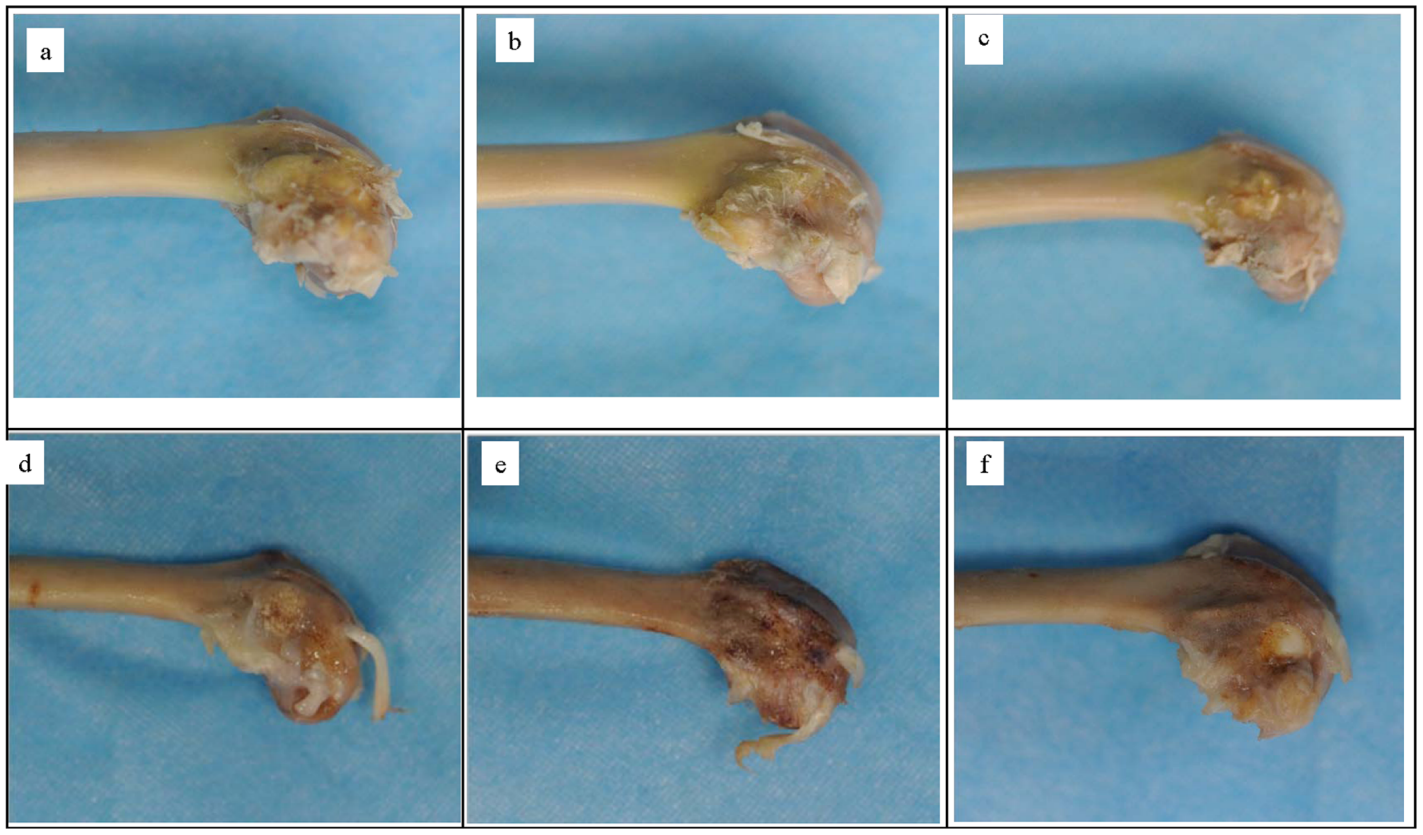
Macroscopic evaluation of CPC-BG composite and CPC specimens implanted into bone defects of rabbits for 4 and 12 weeks. (a) CPC+10% BG for 4 weeks. (b) CPC+20% BG for 4 weeks. (c) CPC for 4 weeks. (d) CPC+10% BG for 12 weeks. (e) CPC+20% BG for 12 weeks. (f) CPC specimens for 12 weeks.

After 4 weeks’ implantation, macroscopic observations of CPC and CPC-BG composites implanted into the bone defects of rabbit lateral femoral condyles showed that the implants exhibited no foreign body reaction, no inflammation and no necrosis in vivo and were incorporated well with surrounding tissue. All CPC-BG composite specimens were covered with a tissue layer that was indistinguishable from surrounding tissue (Figure 9a, b). Conversely, CPC specimens were covered with a thinner tissue layer that could be distinguished by macroscopic evaluation (Figure 9c). With the increase of the implantation period to 12 weeks, the volume of CPC-BG composite specimens decreased accompanied by a simultaneous bone ingrowth from the periphery inwards due to the degradation of the CPC-BG composite. The boundaries between normal surrounding tissue and the specimens were indistinct, and the newly formed bone could not be distinguished from normal bone (Figure 9d, e). Conversely, CPC specimens did not show observable variations in size from the original implantation after 12 weeks of degradation and the bone ingrowth mainly occurred at the native bone margins and the defect periphery (Figure 9f).

### Micro-CT Analysis

**Figure 10:**
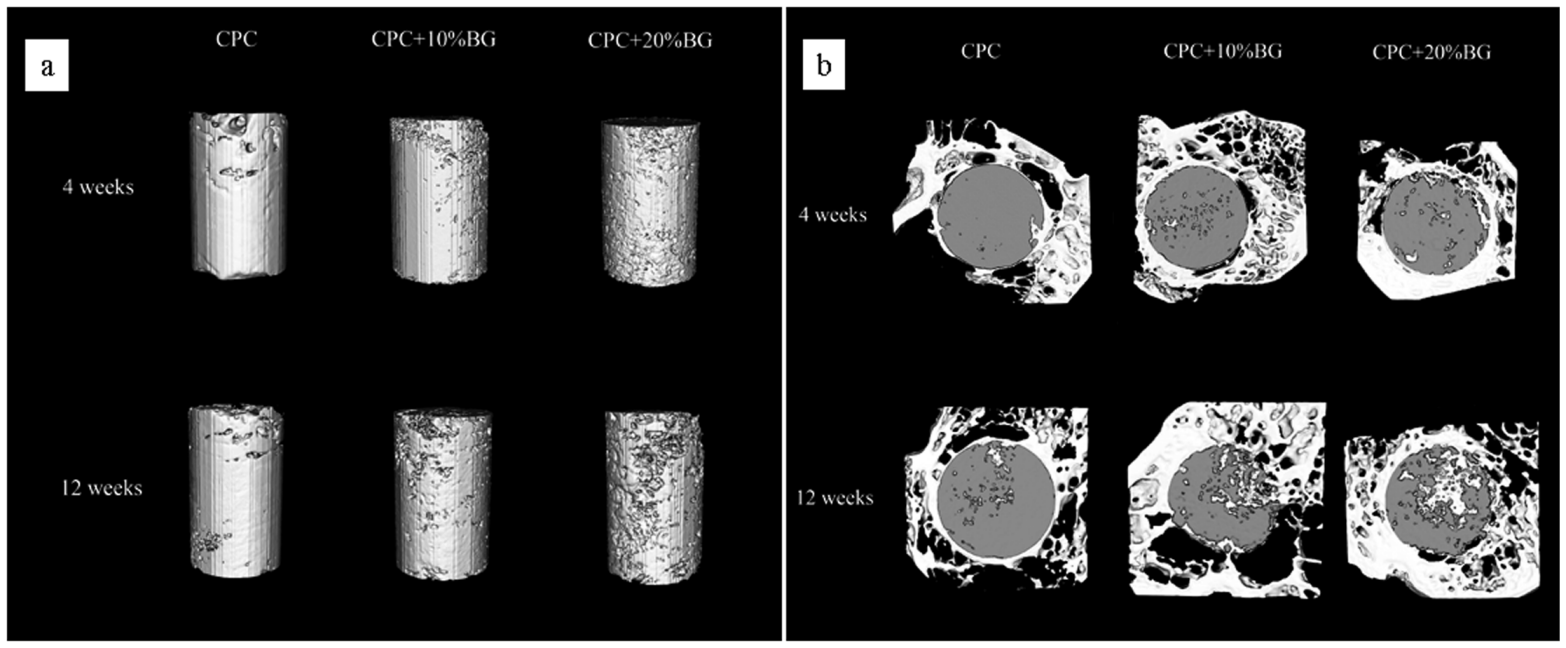
Three-dimensional reconstruction using micro-CT analysis. (a) residual material of the CPC-BG composite and CPC. (b) cross-sectional images of rabbit femur after implantation for 4 and 12 weeks.

**Figure 11:**
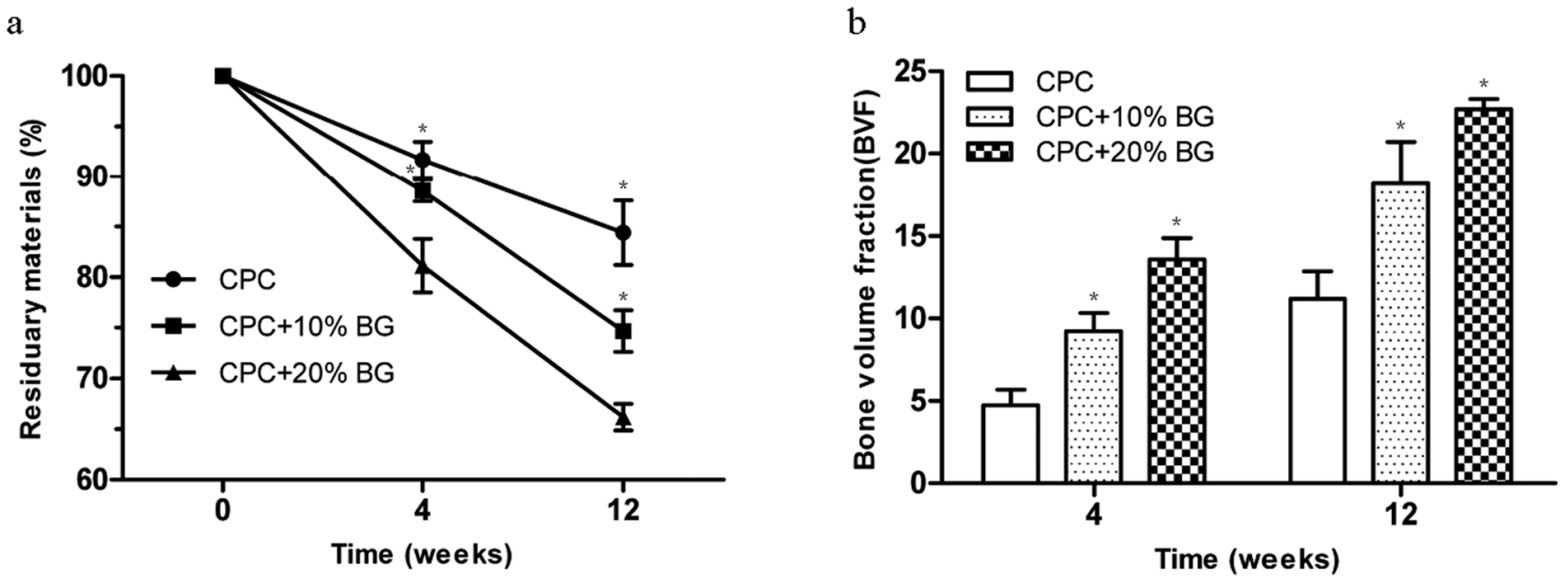
Quantitative analysis of residual material and new bone formation from micro-CT images. (a) residual material. (b) new bone formation for 4 and 12 weeks.

3D reconstruction images of residual material of the CPC and CPC-BG composite specimens after implantation for 4 and 12 weeks were adopted to assess the in vivo resorption of the implants (Figure 10a). After a prolonged implantation time from 4 to 12 weeks, the surface morphologies of the CPC-BG composite specimens exhibited many differences from their original appearances. After 12 weeks’ implantation, a porous surface structure was obtained. With increased implantation time, the pore size formed by degradation became larger and the volumes of the CPC-BG composite specimens decreased. Conversely, the CPC specimens showed rare variations in appearance and volume and pore formation was mainly found at the outermost edge of the implants at 12 weeks after implantation. Figure 10b displays the bone ingrowth into the implants at 4 to 12 weeks. At 4 weeks, there was a small amount of newly formed bone at the interface of the CPC-BG composite specimens, and more extensive bone ingrowth occurred throughout the cross-section of specimens at 12 weeks after implantation. However, bone formation in the CPC group was mostly observed at the defect periphery at 4 weeks and only a small amount of new bone tissue was found within the implants 12 weeks after implantation (Figure 10b). From 4 to 12 weeks, the RMVF of CPC-BG (20%) composite specimens decreased from 81.16±2.66% to 66.18±1.32% whereas the RMVF of CPC remained at 84.42±3.20% after 12 weeks of implantation, revealing that the in vivo degradation of the CPC-BG composites was much higher than that of CPC (*p*<0.05) (Figure 11a). The BVF was applied to evaluate the newly formed bone more precisely (Figure 11b). The BVF of each CPC-BG composite specimen was significantly higher than the BVF of each CPC specimen at both 4 and 12 weeks (*p*<0.05).

These results confirmed that CPC-BG showed excellent biocompatibility, degradability and osteogenesis, and CPC-BG exhibited greater bone-forming efficiency than CPC.

### Histological Analysis

**Figure 12:**
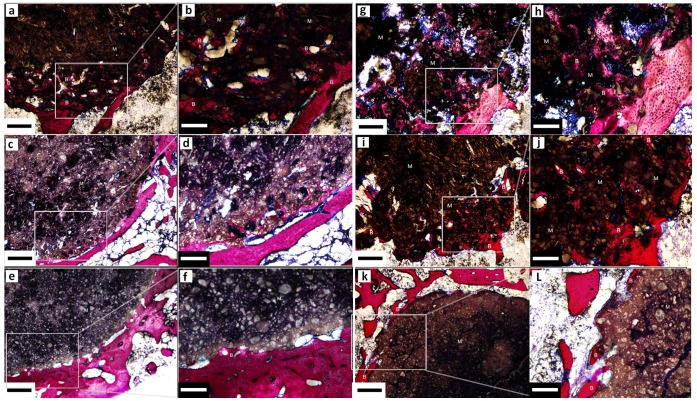
Van Gieson’s-stained sections of CPC-BG composite and CPC specimens that were harvested at 4 (a–f) and 12 (g–l) weeks after implantation. (a, b) CPC+20% BG for 4 weeks. (c, d) CPC+10% BG for 4 weeks. (e, f) CPC for 4 weeks. (g, h) CPC+20% BG for 12 weeks. (i, j) CPC+10% BG for 12 weeks. (k, l) CPC for 12 weeks. M: materials. B: bone formation. Scale bar: 400 µm (black), 160 µm (white).

After 4 weeks’ implantation, the CPC-BG composite implant was encapsulated by the surrounding bone tissue and the boundary between the implant and host bone was detectable. The material had started to degrade from the edge of the implant, and new bone tissues had grown into the pores formed by the degradation of the CPC-BG composite implant. The new bone was in direct contact with the surface of the implant (Figure 12a–d). After 12 weeks’ implantation, bone ingrowth had occurred in many areas of the implant and the amount of newly formed bone in those defects had increased dramatically together with the resorption of the CPC-BG composite implant. Direct contact between the new bone and the CPC-BG composite implant increased from 4 weeks to 12 weeks (Figure 12g–j). For CPC, resorption of CPC rarely occurred, and new bone tissue formed only rarely at the interface of the implant after 4 weeks (Figure 12e, f). After 12 weeks’ implantation, there was only marginal degradation in the CPC implant and no fibrous layer appeared between the bone and the implant surface (Figure 12k, l), which is in accordance with the results of the macroscopic evaluation and the micro-CT analysis. These results confirmed that the CPC-BG composite showed excellent biocompatibility, biodegradability and osteogenesis, and that the CPC-BG exhibited significant advantages over CPC.

## Discussion

A major drawback of orthopedic implant materials such as hydroxyapatie (HA) ceramic in current use is their hardened form, which requires the surgeon to drill the surgical site around the graft or to carve the implant into the desired shape and often leads to increased bone loss, trauma and surgical time [21]. With the emergence of minimally invasive surgery techniques, materials with self-setting properties have drawn more attention [22], [23]. In the present study, a novel injectable CPC-BG composite has been developed that can be mixed with the cement liquid (potassium phosphate buffers) to form a paste that can be applied to fill irregular bone cavities during surgery via injection especially where shaping and contouring for esthetics are needed. The material exhibited a retarded setting time and, consequently, improved injectability due to the addition of BG as compared with CPC paste. However, in clinical applications, the cement must be applied within the initial setting time and be extruded during the operation. Compared with CPC paste, which sets in <10 min, CPC-BG composite paste’s extra setting time (21 min for 10 wt% BG or 25 min for 20 wt% BG) gives an advantage to surgeons or dentists by allowing more time for the operation before the paste set.

As the cement is used for bone repair, the mechanical properties of the hardened cement are another important index [24]. Many researchers have worked to improve the mechanical strength of CPC [7], [18], [25]. For instance, the mechanical strength of DCPD/C_3_S cement is improved by mixing CaHPO_4_·2H_2_O (DCPD) and Ca_3_SiO_5_ (C_3_S) mainly due to the filler effect of the calcium silicate’s hydrated phase and the chemical interaction between the CPC matrix and C_3_S particles. However, the setting time of the DCPD/C_3_S cement with 40 wt% C3S was prolonged to approximatlely 60 min with increased C_3_S amount, which would enhance the chance of the cement being washed out by physiological liquid during clinical application [26]. In the present study, the compressive strength of CPC-BG composite specimens was significantly higher than the compressive strength of CPC, and the CPC-BG composite specimens with 20 wt% BG possessed the highest compressive strength, at approximaltely 26 MPa and 40 MPa after setting for 1 and 7 days, respectively. This high strength may be due to the lower setting rate and consequently more compact microstructure of the CPC-BG composite (Figure 2). Moreover, Si ions tend to inhibit grain the growth of HA crystals [27], which further contributes to the tendency of the CPC-BG composite to form a compact and homogeneous microstructure. However, the exact interaction mechanism between CPC and BG need to be further illuminated.

For bioactive substitution materials, it is important to induce a bone-like apatite layer on the surface that can form chemical bonds to bone tissue at the early stage of the implantation [28]. Bioglass has been reported to form bone-like apatite on its surface after being soaked in SBF [29]. In the present study, the number and grain size of apatite aggregates on CPC and CPC-BG composite surfaces increased with prolonged immersion time. However, the number of apatite aggregates on the surface of the CPC-BG composite was larger than the amount on the CPC surface after immersion for 7 and 14 days. Moreover, this apatite layer grew more densely when there was a greater content of BG in the CPC-BG composite, indicating that the bioactivity of the CPC-BG composite was improved. These results suggest that the addition of BG could result in a bioactive composite with controllable bioactivity. It is well known that the formation of apatite on the surface of CPC mainly relies on supersaturation of Ca^2+^ and PO_4_^3−^ ions and that the process of deposition proceeding at a low rate leads to absent homogeneous apatite layer formation at the early stages of the implantation [30]. With the addition of BG in the composite, a SiO^2−^ rich gel layer forms on the surface of the BG and plays an important role in the formation of a CaO-P_2_O_5_-rich film, which provides favorable sites for nucleation of apatite crystals that form the apatite layer in the simulated body environment. As the content of BG increases, the CPC-BG composite provides more nucleation sites for the apatite crystals, resulting in the formation of a homogeneous apatite layer on the surface of the CPC-BG composite. The assumption that the CPC-BG composite forms a stronger bond with the surrounding bone tissue than CPC needs to be further confirmed by in vivo studies.

The biomaterial should be degradable and gradually replaced by newly formed bone tissue [31]. Proper degradation in a physiological environment is one of the most important characteristics of in bone repair applications. The degradation rate of CPC, which is composed of TECP and DCPA, is slow [25]. In the present study, the degradation rate of CPC-BG composite was significantly faster than the degradation of CPC, which can be attributed to the higher solubility of BG. In addition, the degradation rate of the CPC-BG composite can be adjusted by controlling the BG content.

The cellular responses to biomaterials can be influenced by surface characteristics of the biomaterials in vitro. Ideally, bioactive materials should interact actively with cells and stimulate cell growth [32]. A first effort to culture rat osteoblasts on the novel CPC-BG composite was performed to evaluate cell attachment, proliferation and differentiation. Because the cell attachment stage is the initial stage of interaction between the cells and the biomaterial, its quality will directly affect cell growth, morphology, proliferation and differentiation [34]. Cells adhered better to the surface of the CPC-BG composite specimen than CPC after 8 h, indicating that the CPC-BG composite was superior over CPC in promoting cellular attachment. Furthermore, cells adhered best to the CPC-BG composite with 20 wt% BG, suggesting that BG played an important role in promoting cellular attachment. It has been known that surface silanols (Si-OH) of BG, which generate a high negative-surface-charge density, contribute to the strong irreversible adsorption of serum proteins [35]. The enhancement of cell attachment was mostly likely associated with the preferential adsorption of serum proteins such as fibronectin onto BG, an intermediary step preceding cell attachment to the biomaterial surface [36].

Both CPC and BG have been shown to be biocompatible in previous in vitro and in vivo studies [33]. The novel CPC-BG composite exhibited a positive cellular behavior and was shown to be cyto-compatible with no obvious negative effects on cellular viability.

ALP activity was routinely used as an early marker of osteoblast differentiation in vitro. Fetal osteoblastes were shown to differentiate in a 45S5 Bioglass® conditioned medium in the absence of osteogenic supplements [12]. 45S5 BG exhibited a significant effect on the early differentiation of marrow stromal cells into osteoblast-like cells [10]. Interestingly, it has been reported that this effect on differentiation of rat MSCs, was not observed in human MSCs when cells were grown on BG or with BG dissolution products [37]. Similarly, additional findings showed that BG-supplemented materials did not affect ALP of human MSCs in vitro but they did elicit a marked increase in bone formation in vivo where a complex mixture of cells and growth factors was present. This difference indicates that BG supports bone formation through a more complex mechanism than direct stimulation of MSC differentiation [38]–[40]. Recently, it was reported that PLGA-S-BG composites supported BMP-mediated osteogenesis of human BMSCs, which reflects the good osteoinductive properties of BG [41]. In the present study, our results showed that the CPC-BG composite promoted cellular differentiation and possessed excellent bioactivity when BG was added as a constituent. Thus, to better understand the effect of BG on bone cells, a more thorough study on the human MSC response to BG or BG-supplemented materials in vitro is necessary.

Biocompatibility is a factor relevant to the response of cells that are in contact with the biomaterial, and it has been reported that the surface of biomaterials may affect the behavior and morphology of cells cultured on their surface [42]. SEM results for cell morphology confirmed that cells attached and spread on the surfaces of both the CPC-BG composite and CPC. The observed cell-to-cell junctions revealed that both the CPC-BG composite and CPC were suitable for cell attachment and growth. At day 4, the number of cells on the CPC-BG composite appeared to be more than the number on the CPC specimens, which can be attributed to strong irreversible adsorption of serum proteins onto BG.

Moreover, cellular responses to biomaterials, such as cell attachment, proliferation and differentiation, depend not only on the surface morphology but also on the chemical composition of the biomaterial [43], which plays a crucial part in determining the cell-material interaction for biomaterials by influencing the quantity of ions released from the biomaterial [44]. Previous studies have demonstrated that ion dissolution products containing Ca and Si that were released from BG can stimulate cell attachment, proliferation, differentiation and mineralization [10]–[14]. In the present study, dissolution of the CPC-BG composite provided a Ca- and Si-rich environment to stimulate cell growth, proliferation and differentiation.

In the in vivo study, the macroscopic evaluation results showed that both the CPC-BG composite and CPC implants exhibited no obvious inflammatory response, rejection or necrosis in the adjacent host tissue and they incorporated well with the surrounding tissue. With the prolonged time to 12 weeks, the boundaries between CPC-BG composite specimens and normal surrounding tissue were indistinct due to the degradation of specimens and subsequent ingrowth of new bone. Conversely, the CPC specimens exhibited few variations in size after 12 weeks implantation. It is well known that resorption of the bone-substitute material is required in the replacement of bone tissue because bone ingrowth into the defect area requires the liberation of the space [45]. In this study, the precise evaluation of in vivo degradation and newly formed bone was confirmed with micro-CT analysis. The in vivo resorption increased with prolonged implantation time. There was remarkably higher in vivo degradation of CPC-BG composites, which substantially influenced bone formation. As the implantation time increased, new bone was regenerated and gradually penetrated into the implant accompanied by the resorption of the CPC-BG composite implant. It is believed that chemical dissolution was the main way of resorption for the implant during the early period of implantation because it changed and enlarged the microstructure of the implant, which could facilitate cell-mediated resorption later on. The increased degradation of the CPC-BG composite implant might be related to the increased dissolution of BG after contact with fluids [40]. Additionally, ionic dissolution products of BG have been reported to beneficially affect osteogenesis by formation of a hydroxycarbonate apatite (HCA) layer and promotion of bone growth [11], [12]. Moreover, it has been suggested that BG has a stimulatory effect on neovascularization [39], [40], which, together with the osteopromotive properties of BG, might further influence bone formation when using a CPC-BG composite as the bone-substitute material. BVF gradually increased while the volume of the CPC-BG composite specimens continued to decrease over time, which indicates that cell-mediated resorption occurred. Direct and intimate contact appeared at the interfaces of both CPC-BG composite and CPC specimens. However, a quantitative analysis showed that the BVF values for the CPC-BG composite specimens were greatly higher than the values for CPC. Moreover, more extensive bone ingrowth occurred throughout the cross-sections of the CPC-BG composite specimens, which indicates that more effective osteogenesis and oseointegration had occurred at the defect area; these indicators are considered to be critical to firmly anchor the implant in place [46]. Histological evaluation revealed that the CPC-BG composite specimens were encapsulated by the surrounding bone tissue, and the new bone was in direct contact with the implant 4 week after implantation. New bone tissues formation increased considerably both in and along the implant together with the resorption of the CPC-BG composite implant 12 weeks after implantation. The implant of CPC-BG composite implant formed tight and direct bonding with the surrounding host bone without the intervention of soft tissue, in accordance with macroscopic evaluation and micro-CT results. These results confirmed that the CPC-BG composites exhibited not only faster biodegradability but also enhanced and more effective osteogenesis and osteointegration at the defect area; these are significant advantages over CPC. However, our study was carried out under optimal conditions. Considering the differences in bone metabolism for healthy bone compared with those under compromised conditions, the biological performance of CPC-BG composite should be tested for compromised conditions such as osteoporosis. Moreover, long-term studies of CPC-BG composites for bone regeneration should be carried out. As an ideal implanted biomaterial candidate for bone regeneration, the CPC-BG composites discussed herein presented good biocompatibility and osteoconductive properties and induced bone ingrowth into the implant. In addition, the material was shown to be resorbable and it is replaced by new bone in a creeping substitution manner. Finally, the material can be handled as a paste and set in situ within a comfortable time. Our findings suggested that CPC-BG is a potential bioactive composite material for bone regeneration in future clinical situations.

### Conclusions

A novel injectable CPC-BG composite was first fabricated and characterized by incorporating BG into CPC and showed a prolonged setting time with improved injectability. The mechanical strength of the CPC-BG composite was significantly enhanced over CPC. Furthermore, incorporation of BG into the CPC appeared to significantly improve the bioactivity and degradability. The CPC-BG composite promotes the attachment, proliferation and differentiation of rat osteoblasts and exhibits excellent biocompatibility with no negative effects on cell morphology or viability. Macroscopic observations of CPC-BG composite implants exhibited no obvious inflammatory response, rejection or necrosis, and the implants incorporated well with the surrounding tissue in vivo. Micro-CT and histological evaluations confirmed that the CPC-BG composite implants exhibited more effective osteogenesis and osteointegration at the defect area than CPC with good biocompatibility and biodegradability. In conclusion, this novel injectable biomaterial with improved properties by incorporation of BG into CPC exhibits promising prospects for bone regeneration.

## References

[1] Wu F, Wei L, Guo H, Liu CS (2008) Self-setting bioactive calcium-magnesium phosphate cement with high strength and degradability for bone regeneration. Acta Biomaterialia 4: 1873–1884. 18662897 10.1016/j.actbio.2008.06.020

[2] Komaki H, Tanaka T, Chazono M, Kikuchi T (2006) Repair of segmental bone defects in rabbit tibiae using a complex of beta-tricalcium phosphate, type I collagen, and fibroblast growth factor-2. Biomaterials 27: 5118–5126. 16769112 10.1016/j.biomaterials.2006.05.031

[3] Link DP, van den Dolder J, van den Beucken JJ, Wolke JG, Mikos AG, et al (2008) Bone response and mechanical strength of rabbit femoral defects filled with injectable CaP cements containing TGF-b1 loaded gelatin microspheres. Biomaterials 29: 675–682. 17996293 10.1016/j.biomaterials.2007.10.029

[4] Brown WE, Chow LC (1986) A new calcium phosphate water setting cement. In: Brown PW, editor. Cements research progress. Westerville, OH: American Ceramic Society p. 352–379.

[5] Julien M, Khairoun I, LeGeros RZ, Delplace S, Pilet P, et al (2007) Physico-chemical–mechanical and in vitro biological properties of calcium phosphate cements with doped amorphous calcium phosphates. Biomaterials 28: 956–965. 17123598 10.1016/j.biomaterials.2006.10.018

[6] Friedman CD, Costantino PD, Takagi S, Chow LC (1998) Bonesource hydroxyapatite cement: a novel biomaterial for craniofacial skeletal tissue engineering and reconstruction. J Biomed Mater Res 43B: 428–432. 10.1002/(sici)1097-4636(199824)43:4<428::aid-jbm10>3.0.co;2-0 9855201

[7] Moreau JL, Xu HHK (2009) Mesenchymal stem cell proliferation and differentiation on an injectable calcium phosphate- Chitosan composite scaffold, Biomaterials. 30: 2675–2682. 10.1016/j.biomaterials.2009.01.022 PMC2662619 19187958

[8] Xu HHK, Zhao L, Detamore MS, Takagi S, Chow LC (2010) Umbilical cord stem cell seeding on fast-resorbable calcium phosphate bone cement. Tissue engineering: Part A. 16: 2743–2753. 10.1089/ten.tea.2009.0757 PMC2928047 20388037

[9] Bodde E, Boerman O, Russel F, Mikos A, Spauwen P, et al (2008) The kinetic and biological activity of different loaded rhBMP-2 calcium phosphate cement implants in rats. J Biomed Mater Res A 87A: 780–791. 10.1002/jbm.a.31830 18200544

[10] Bosetti M, Cannas M (2005) The effect of bioactive glasses on bone marrow stromal cells differentiation. Biomaterials 26: 3873–3879. 15626435 10.1016/j.biomaterials.2004.09.059

[11] Heikkila JT, Mattila KT, Andersson OH, Knuuti J, Yli-Urpo A, et al (1995) Behavior of bioactive glass in human bone. Bioceramics 8: 35–41.

[12] Tsigkou O, Jones JR, Polak JM, Stevens MM (2009) Differentiation of fetal osteoblasts and formation of mineralized bone nodules by 45S5 Bioglass® conditioned medium in the absence of osteogenic supplements. Biomaterials 30: 3542–3550. 19339047 10.1016/j.biomaterials.2009.03.019

[13] Jell G, Notingher I, Tsigkou O, Notingher P, et al (2008) Bioactive glass-induced osteoblast differentiation: A noninvasive spectroscopic study. J Biomed Mater Res A 86A: 31–40. 10.1002/jbm.a.31542 17941016

[14] Christodoulou I, Buttery LD, Saravanapavan P, Tai G, Hench LL, et al (2005) Dose- and time-dependent effect of bioactive gel-glass ionic-dissolution products on human fetal osteoblast-specific gene expression. J Biomed Mater Res B Appl Biomater 74B: 529–537. 10.1002/jbm.b.30249 15889438

[15] Radin S, Reilly G, Bhargave G, Leboy PS, Ducheyne P (2005) Osteogenic effects of bioactive glass on bone marrow stromal cells. J Biomed Mater Res A 73: 21–29. 15693019 10.1002/jbm.a.30241

[16] Renno ACM, van de Watering FCJ, Nejadnik MR, Crovace MC, et al (2013) Incorporation of bioactive glass in calcium phosphate cement: An evaluation. Acta Biomaterialia 9: 5728–5739. 23159565 10.1016/j.actbio.2012.11.009

[17] Guo H, Su JC, Wei J, Kong H, Liu CS (2009) Biocompatibility and osteogenicity of degradable Ca-deficient hydroxyapatite scaffolds from calcium phosphate cement for bone tissue engineering. Acta Biomaterialia 5: 268–278. 18722167 10.1016/j.actbio.2008.07.018

[18] Huan ZG, Chang J (2009) Calcium-phosphate-silicate composite bone cement: self-setting properties and in vitro bioactivity. J Mater Sci: Mater Med 20: 833–841. 19034622 10.1007/s10856-008-3641-9

[19] Kokubo T, Takadama H (2006) How useful is SBF in predicting in vivo bone bioactivity. Biomaterials 27: 2907–2915. 16448693 10.1016/j.biomaterials.2006.01.017

[20] Wada Y, Kataoka H, Yokose S, Ishizuya T, et al (1998) Changes in osteoblast phenotype during differentiation of enzymatically isolated rat calvaria cells. Bone 22: 479–485. 9600781 10.1016/s8756-3282(98)00039-8

[21] Laurencin CT, Ambrosio AMA, Borden MD, Cooper JA (1999) Tissue engineering: orthopedic applications. Annu Rev Biomed Eng 1: 19–46. 11701481 10.1146/annurev.bioeng.1.1.19

[22] Combes C, Tadier S, Galliard H, Girod-Fullana S, et al (2010) Rheological properties of calcium carbonate self-setting injectable paste. Acta Biomaterialia 6: 920–927. 19716448 10.1016/j.actbio.2009.08.032

[23] Chen ZG, Liu HY, Liu X, Cui FZ (2011) Injectable calcium sulfate/mineralized collagen-based bone repair materials with regulable self-setting properties. J Biomed Mater Res A 99: 554–563. 21936045 10.1002/jbm.a.33212

[24] Zhang JT, Tancret F, Bouler JM (2011) Fabrication and mechanical properties of calcium phosphate cements (CPC) for bone substitution. Mater Sci Eng C 31: 740–747.

[25] Lu JX, Wei J, Yan YG, Li H, et al (2011) Preparation and preliminary cytocompatibility of magnesium doped apatite cement with degradability for bone regeneration. J Mater Sci: Mater Med 22: 607–615. 21258847 10.1007/s10856-011-4228-4

[26] Lin Q, Lan XH, Li YB (2010) Anti-washout carboxymethyl chitosan modified tricalcium silicate bone cement: preparation, mechanical properties and in vitro bioactivity. J Mater Sci: Mater Med 21: 3065–3076. 20890641 10.1007/s10856-010-4160-z

[27] Huan ZG, Chang J (2009) Novel bioactive composite bone cements based on the β-tricalcium phosphate-monocalcium phosphate monohydrate composite cement system. Acta Biomaterialia 5: 1253–1264. 18996779 10.1016/j.actbio.2008.10.006

[28] Kobayashi M, Nakamura T, Shinzato S, Mousa WF, Nishio K, et al (1999) Effect of bioactive filler content on mechanical properties and osteoconductivity of bioactive bone cement. J Biomed Mater Res 46: 447–457. 10398005 10.1002/(sici)1097-4636(19990915)46:4<447::aid-jbm2>3.0.co;2-p

[29] Miguel BS, Kriauciunas R, Tosatti S, Ehrbar E, et al (2010) Enhanced osteoblastic activity and bone regeneration using surface-modified porous bioactive glass scaffolds. J Biomed Mater Res A 94A: 1023–1033. 10.1002/jbm.a.32773 20694969

[30] He Q, Chen H, Huang L, Dong J, Guo D, et al (2012) Porous surface modified bioactive bone cement for enhanced bone bonding. PLoS One 7: e42525. 22905143 10.1371/journal.pone.0042525 PMC3414445

[31] Hu GF, Xiao LW, Fu H, Bi DW, et al (2010) Study on injectable and degradable cement of calcium sulphate and calcium phosphate for bone repair. J Mater Sci: Mater Med 21: 627–634. 19823918 10.1007/s10856-009-3885-z

[32] Chen QZ, Efthymiou A, Salih V, Boccaccini AR (2008) Biogalss® -derived glass-ceramic scaffolds: Study of cell proliferation and scaffold degradation in vitro. J Biomed Mater Res A 84A: 1049–1060. 10.1002/jbm.a.31512 17685403

[33] Saboor A, Rabiee M, Mutarzadeh F, Sheikhi M, Tahriri M, et al (2009) Synthesis, characterization and in vitro bioactivity of sol-gel-derived SiO_2_-CaO-P_2_O_5_-MgO bioglass. Mater. Sci 29: 335–340.

[34] Low SP, Williams KA, Canham LT, Voelcker NH (2006) Evaluation of mammalian cell adhesion on surface modified porous silicon. Biomaterials 27: 4538–4546. 16707158 10.1016/j.biomaterials.2006.04.015

[35] Manitha B, Nair HK, Varma AJ (2009) Triphasic ceramic coated hydroxyapatite as a niche for goat cell-derived osteoblasts for bone regeneration and repair. J Mater Sci: Mater Med 20: S251–258. 18853240 10.1007/s10856-008-3598-8

[36] Lee MH, Ducheyne P, Lynch L, et al (2006) Effect of biomaterial surface properties on fibronectin-alpha(5)beta(1) integrin interaction and cellular attachment. Biomaterials 27: 1907–1916. 16310247 10.1016/j.biomaterials.2005.11.003

[37] Reilly GC, Radin S, Chen AT, Ducheyne P (2007) Differential alkaline phosphatase responses of rat and human bone marrow derived mesenchymal stem cells to 45S5 bioactive glass. Biomaterials 28: 4091–4097. 17586040 10.1016/j.biomaterials.2007.05.038 PMC2699612

[38] Yao J, Radin S, Reilly G, Leboy P, Ducheyne P (2005) Solution-mediated effect of bioactive glass in poly(lactic-co-glycolic acid)-bioactive glass composites on osteogenesis of marrow stromal cells. J Biomed Mater Res 75A: 794–801. 10.1002/jbm.a.30494 PMC1432094 16138322

[39] Leach JK, Kaigler D, Wang Z, Krebsbach PH, Mooney DJ (2006) Coating of vegf-releasing scaffold with bioactive glass for angiogenesis and bone regeneration. Biomaterials 27: 3249–3255. 16490250 10.1016/j.biomaterials.2006.01.033

[40] Hoppe A, Guldal NS, Boccaccini AR (2011) A review of the biological response to ionic dissolution products from bioactive glasses and glass-ceramics. Biomaterials 32: 2757–2774. 21292319 10.1016/j.biomaterials.2011.01.004

[41] Pamula E, Kokoszka J, Cholewa-kowalska K, Laczka M, Kantor L, et al (2011) Degradation, bioactivity, and osteogenic potential of composites made of PLGA and two different sol-gel bioactive glasses. Ann Biomed Eng. 39: 2114–2129. 10.1007/s10439-011-0307-4 PMC3127015 21487840

[42] Lee JY, Kang BS, Hicks B, Chancellor TF, Chu BH, et al (2008) The control of cell adhesion and viability by zinc oxide nanorods. Biomaterials 29: 3743–3749. 18550161 10.1016/j.biomaterials.2008.05.029

[43] Chou SY, Cheng CM, LeDuc PR (2009) Composite polymer systems with control of local substrate elasticity and their effect on cytoskeletal and morphological characteristics of adherent cells. Biomaterials 30: 3136–3142. 19299009 10.1016/j.biomaterials.2009.02.037

[44] Wu C, Ramaswmy Y, Zhu YF, Zheng R, Appleyard R, et al (2009) The effect of mesoporous bioactive glass on the physiochemical, biological and drug-release properties of poly(DL-lactide-co-glycolide) films. Biomaterials 30: 2199–2208. 19203787 10.1016/j.biomaterials.2009.01.029

[45] van de Watering FCJ, van den Beucken JJJP, Walboomers XF, Jansen JA (2012) Calcium phosphate/poly (D, L-lactic-co-glycolic acid) composite bone substitute materials: evaluation of temporal degradation and bone ingrowth in a rat critical-sized cranial defect. Clin Oral Implants Res 23: 151–159. 21631594 10.1111/j.1600-0501.2011.02218.x

[46] Hennessy KM, Clem WC, Phipps MC, Sawyer AA, Shaikh FM, et al (2008) The effect of RGD peptides on osseointegration of hydroxyapatite. Biomaterials 29: 3075–3083. 18440064 10.1016/j.biomaterials.2008.04.014 PMC2465812

